# Determinants of unmet need for family planning among currently married women in Dangila town administration, Awi Zone, Amhara regional state; a cross sectional study

**DOI:** 10.1186/s12978-015-0038-3

**Published:** 2015-05-13

**Authors:** Ewnetu Genet, Gedefaw Abeje, Tadese Ejigu

**Affiliations:** Amhara Development Association, Bahir Dar Ethiopia, Ethiopia; School of Public Health, College of Medicine and Sciences, Bahir Dar University, Bahir Dar, Ethiopia

**Keywords:** Unmet need, Family planning, Dangila town, Awi Zone

## Abstract

**Background:**

Unmet need for family planning is a major problem of developing countries. Evidences about unmet need for family planning and associated factors are not enough in Dangila town. Therefore, this study was done to assess the magnitude and determinants of unmet need for family planning among currently married women in Dangila town.

**Methodology:**

Community based cross sectional study design was used to collect data from a total of 551 currently married women from February to March 2014. Data were collected using pretested structured interviewer administered questionnaire after written consent was obtained from respondents. Collected data were edited, coded, and entered to SPSS version 16.0. Bivariate and multivariable logistic regression analyses were done to identify determinants of unmet need for family planning.

**Results:**

This study revealed that 17.4 % of married women had unmet need for family planning. In this study, women who were housewife/farmers were about 7 [OR = 6.81 (1.91–24.29)] times more likely to have unmet need compared to employed women. Women who were not counseled about family planning by health workers [OR = 6.76 (3.17–14.42)], women whose partner had non-supportive attitude for family planning use [OR = 3.34 (1.26–8.90)] and rural women [OR = 17.65 (4.35–71.67)] were also more likely to have unmet need for family planning. About 33 %, 32 %, 23.5 % and 11.8 % of women mentioned less perceived risk of pregnancy due to breast feeding, fear of side effects, partner opposition and religious prohibition respectively as reasons for not using contraceptives at the time of interview.

**Conclusions:**

The level of unmet need for family planning in the study area is still high compared to the target set (10 %) in the national family planning guide plan of Ethiopia to be achieved by the end of 2015. Therefore, it is important to strengthen counseling and partner involvement in Dangila town to reduce unmet need for family planning.

## Background

Family planning has many potential benefits. It reduces poverty, maternal and child mortality; empowers women by lightening the burden of excessive childbearing and it reduces environmental degradation by stabilizing the population of the planet [1, 2].

Unintended pregnancy related to unmet need is a worldwide problem that affects women and their families and societies at large. About 40 % of all births that occurred globally in 2012 were unwanted posing hardships for families and jeopardizing the health of millions of women and children [3]. Serving all women in developing countries who currently have an unmet need for modern methods would prevent an additional 54 million unintended pregnancies, including 21 million unplanned births, 26 million abortions (of which 16 million would be unsafe) and seven million miscarriages; this would also prevent 79,000 maternal deaths and 1.1 million infant deaths [4]. In sub-Saharan Africa, 25 % of women of reproductive age who are married or in union have an unmet need for family planning [5].

The contraceptive prevalence rate in Ethiopia was about 29 % in 2011. The few surveys conducted on issues related to unmet need for FP suggested that unwanted pregnancy and unsafe abortion are main causes of maternal mortality in Ethiopia [6]. According to the 2011 Ethiopian demographic and health survey, the level of unmet need in Ethiopia was 25.3 %. This study also showed that unmet need for family planning in Amhara region was about 22 % [6].

In a study conducted in Harar, 33.3 % the pregnant women reported that their most recent pregnancies were unintended. Of these, 50 % had unintended childbirths and the other 50 % ended with induced abortion [7]. A study conducted in Butajira on determinants of low family planning use showed that unmet need for limiting was lowest in urban Butajira and highest in rural Butajira. Mean while unmet need for spacing was much higher in rural areas compared to urban Butajira [8].

To address problems associated with unmet need for family planning evidences about the magnitude and determinants of unmet need are essential. However, these evidences are not enough in Dangila town. This study was therefore done to determine the level of unmet need for family planning and associated factors among married reproductive age women in Dangila town.

## Methodology

A community based cross-sectional study was conducted in Dangila town administration from February to March 2014. The town administration is subdivided into five rural and five urban Kebeles (smallest administrative unit).

All currently married reproductive age (15–49) women who were living in Dangila town administration were included in this study.

Sample size was calculated using single population proportion formula. Assuming 22.1 % unmet need 5 % margin of error, 95 % confidence level and design effect of 2, the total sample size was 556. Households with married reproductive age women were listed out from family folder of health extension workers (HEW). Study participants were allocated proportionally to each kebele based on number of married reproductive age women in selected kebeles. Then households with reproductive age women were selected by using simple random sampling technique. Finally, reproductive age women in the selected household were interviewed. We used lottery method to select one reproductive age women when there were more than one reproductive age women in the selected household.

Five diploma nurses collected the data using structured, pretested interviewer administered questionnaire prepared in Amharic (the local language). Two days training was given for data collectors and supervisors.

Data collected each day were checked visually for completeness by the supervisors and the principal investigator. Then, the investigators entered the data in to SPSS version 16.0 for cleaning and analysis. Bivariate and multivariable analyses were done to identify factors associated with unmet need for family planning. Odds ratio with 95 % CI and p-values were used to declare the presence of statistical association.

In this study, a woman was said to have unmet need for family planning if she was fecund but do not want more child or if she want to wait at least two years before having another child but are not using any contraception. A woman whose pregnancy is unwanted or mistimed because she was not using contraceptive is also classified as a woman with unmet need for family planning.

Ethical clearance was obtained from Bahir Dar University, College of medicine and health science. Letter of permission to conduct the study was granted from Amhara Regional Health Bureau (ARHB) and Dangila town Administration Health Office. Written informed consent was obtained from the study participants after explaining the purpose of the study.

## Result

### Socio-demographic characteristics of the study participants

A total of 551 (423 from urban kebeles & 128 from rural kebeles) women responded for the study making response rate of 99.1 %. Around fifty five percent of respondents were aged 25–34 years. The mean age of women was 29.96 years with standard deviation 6.7 years. Majority of the study participants were from Amhara (95.6 %) ethnic group and Orthodox’s Christian (96.0 %) religion. About 35 % of the respondents were unable to read and write (Table 1).

**Table 1:** Socio-demographic characteristics of currently married women in Dangila town administration, Northwest Ethiopia, February to March, 2014 (*N* = 551)

Variables	Category	Frequency	Percentage
Age in years	15–19	15	2.7
	20–24	91	16.5
	25–29	177	32.1
	30–34	124	22.5
	35–39	75	13.6
	≥40	69	12.5
Educational status	Unable to read and write	190	34.5
	No formal education but can read and write	72	13.1
	Grade 1–8	91	16.5
	Grade 9–12	83	15.1
	Certificate and above	115	20.9
Occupational status	House wife/farmer	271	49.2
	Employed	280	50.8
Educational status of partner	Unable to read and write	76	13.8
	Able to read and write	62	11.3
	Grade 1–8	96	17.4
	Grade 9–12	106	19.2
	Certificate and above	211	38.3
Partner religion	Orthodox christian	530	96.2
	Other	21	3.8
	Government employee	224	40.7
Husband occupation	Private employee	175	31.8
	Farmer	145	26.3
	Other	7	1.3

### Family planning methods use

Out of 551 married women, 402 (79 %) were using contraceptives at the time of interview. Among women who were not using contraceptives, the main reasons were perceived low risk of pregnancy due to breast feeding (32.9 %), fear of side effects (31.8 %), partner opposition (23.5 %) and religious prohibition (11.8 %).

In this study, 393 (71.3 %) women reported that they had heard information about family planning from different sources within the last 12 months. The most frequently mentioned source of information for family planning were television and radio among urban residents and kebele leaders, health extension workers and religious leader for rural residents. In this study, about 95.1 % of women mentioned at least one method of contraception.

### Factors associated with unmet need for FP

After controlling for the possible confounders, residence, occupation, history of counseling about FP by health workers and partner attitude towards FP were found significantly associated with unmet need for FP.

In this study rural women were 17.65 times (AOR = 17.65, 95 % CI: 4.35–71.67) more likely to have unmet need for FP compared to urban women. Housewife/farmers were 6.81 (AOR = 6.81, 95 % CI: 1.91-24.29) times more likely to have unmet need for FP compared to those who were employed. Similarly, women who were not counseled about the use and side effects of contraceptives by health care workers and health development army were 6.76 (AOR = 6.76, 95 % CI: 3.17–14.42) times more likely to have unmet need for FP compared to women who were counseled. Women whose partner was not supportive to contraceptives use were 3.34 (AOR = 3.34, 95 % CI: 1.26–8.90) times more likely to have unmet need for FP compared to those women whose partner was supportive (Table 2).

**Table 2:** Factors associated with unmet need for FP among current married women in Dangila town Administration, February to March 2014

Variables	Unmet need		COR (95 % CI)	AOR (95 % CI)	Over all *p*-value
	Yes	No			
Residence					
Urban	60	363	1.00	1.00	
Rural	36	92	2.37(1.48, 3.80)	17.65(4.35, 71.67)	0.001
Educational status					
Unable to read and write	52	138	14.07(4.28, 46.25)	1.37(0.25, 7.68)	
Able to read and write	25		19.86(5.72, 68.97)	5.01(0.83, 30.14)	
Grade 1–8 completed	13	47	6.22(1.72, 22.56)	0.41(0.06, 2.73)	
Grade 9–12 completed	3	78	1.40(0.28, 7.12)	0.14(0.02, 1.29)	
Certificate and above	3	80 112	1.00	1.00	
Occupation					
House wife /farmer	57	214	1.65(1.05, 2.57)	6.81(1.91, 24.29)	0.003
Employed^a^	39	241	1.00	1.00	
Counseled about FP by health worker and HDA					
Yes	34	359	1.00	1.00	
No	62	96	6.82(4.24, 10.97)	6.76(3.17, 14.42)	0.001
Family planning information given in the last 12 months					
Yes	41	369	0.17(0.11, 0.28)	0.96(0.36, 2.55)	
No	55	86	1.00	1.00	
Partner attitude					
Supportive	20	374	1.00	1.00	
Non-supportive	76	81	17.55(10.14, 30.35)	3.34(1.26, 8.90)	0.016

Employed^a^ (government employee, merchant, private employee)

## Discussion

Reducing the proportion of unmet need for FP has major role in in preventing maternal and child health problems. To reduce the proportion of unmet need for family planning, knowing the current level and its determinants is a prerequisite. This study was conducted to investigate the magnitude and determinants of unmet need for FP among currently married women. The level of unmet need for FP in this study (17.4 %) is lower than the national (25 %) and Amhara regional state level (22.1 %) but slightly higher than the national urban level [6]. This finding had larger discrepancy with similar studies conducted in Bahir district of India, in Butajira district of Ethiopia Kobo and Enemay woreda of Amhara region [8–12].

This large variation may be due to expansion of health facilities and improved access of health services in Ethiopia now. Implementation of health extension program may be also the other reason. On the other hand, the proportion of unmet need for FP in this study is still higher compared to similar studies conducted in Bangladesh and in Iran [13, 14]. This difference might be due to the difference in awareness of people on contraceptives in these countries and the difference in availability of method choice.

In this study rural women were 17.65 (AOR = 17.65, 95 % CI: 4.35–71.67) times more likely to have unmet need for FP compared to urban women. The study also revealed that the level of unmet need for FP among rural residents was twice as high as the level among urban residents (28.1 % and 14.2 % respectively). This discrepancy was almost similar to the national figure which was 15 % and 27.5 % among urban and rural women respectively [6]. But the level of unmet need among urban women in this study was slightly lower than the study conducted in Enemay woreda, East Gojam zone (18.4 %) [12]. These two fold higher unmet needs in rural areas might reflect limited awareness and lower educational status of the rural women in the study area.

On the other hand women who were housewife/farmer were 6.81 (AOR = 6.81, 95 % CI: 1.26–8.90) times more likely to have unmet need compared to those who were employed. The reason for this is that employed women are more likely to have better access for information about FP. Partner support to family planning is very important. This study revealed that women whose partner had non-supportive attitude about contraceptives use were more likely to have unmet need for family planning compared to women whose partners had supportive attitude. This study also showed that women who were not counseled about contraceptives were more likely to have unmet need for FP compared to those women who were counseled. This finding was in line with a study conducted in Mekele city, Tigray [15]. This similarity might be due to use of similar strategies and guide lines to inform the society about FP.

In this study, about 32 % women reported that they were not using contraceptives because of fear of side effects. This finding similar with studies conducted in Enemay, Mojo town and Mekele city of Ethiopia [12, 15, 16]. Another reason mentioned for not using FP in this study was less perceived risk of pregnancy during breast feeding. This finding was supported by a study conducted in Iran [13].

In this study, partner opposition and religious prohibition were mentioned as reasons for not using family planning by 23.5 and 11.8 % of women respectively. These findings were supported by studies conducted in Enemay, and India [9, 12].

This study will enable policy makers to have a look to the determinants of unmet need. It also shows direction for researchers where to focus when studying unmet need for family planning. The findings are very essential to organizations that work on family planning in Dangila town. The limitation of this study is that it covered only small area of Amhara region. This may have effect on its generalizability to the region. Similar socio-demographic characteristics of the study participants also made the confidence interval of the findings wide.

## Conclusions

The proportion of women with unmet need for family planning in Dangila town is still high. In this study, rural women, women who were housewife or farmers, women who were not counseled about use and side effect of contraceptives and women whose partner supportive to family planning use were more likely to have unmet need for FP. The main reasons mentioned for not using contraceptives at the time of interview were fear of side effects, less perceived risk of pregnancy at the time of breast feeding, partner opposition and religious prohibition.
